# Supplementary material for: Are carbon emissions trading and green financial instruments synergistic? -Comprehensive quantitative research based on content analysis
Source: PLoS One. 2024 Mar 7;19(3):e0298601. doi: 10.1371/journal.pone.0298601 (PMC10919601; doi:10.1371/journal.pone.0298601)
Supplement: S1 Data — (ZIP) [file pone.0298601.s001.zip › Data/地级市Z_F工作报告.zip!/╜¡╦╒/─╧═¿╩╨/└┤╫╘╣1⁄2╓┌║┼í╛┬φ┐╦╩2╛▌═°í┐_2003.docx]

各位代表:

　　现在，我代表市十一届人民政府向大会作工作报告，请予审议，并请市政协各位委员和其他列席人员提出意见。

　　一、过去五年的工作回顾

　　本届政府自1998年组成以来，在省委、省政府和市委的领导下，在市人大、市政协的支持与监督下，团结和带领全市干部群众，解放思想，与时俱进，开拓创新，拼搏奋进，圆满实现任期目标。"九五"计划顺利完成，"十五"计划开局良好，经济建设和各项社会事业取得显着成绩。

　　--综合实力显着增强。2002年，全市预计（下同）实现国内生产总值890亿元，比1997年增长65％，年递增10.5％，其间实现了人均国内生产总值超万元的历史性跨越；实现财政收入87.4亿元，同口径比1997年增长119.6％，年递增17％。五年累计完成固定资产投资1272亿元，为前五年的1.52倍。农业结构调整、"海上南通"建设和产业化经营快速推进，农业综合生产能力有较大提高；工业结构发生积极变化，运行质量进一步改善；建筑业持续发展，质量、信誉有了新的提高；房地产、信息、旅游等新兴服务业快速发展，第三产业规模不断扩大。三次产业结构由1997年的21.1：46.7：32.2演进为2002年的15.9：49.3：34.8。

　　--改革开放步伐加快。改革实现新突破。国有经济布局的战略性调整和国有企业的战略性改组取得重大成果，企业改制面全省领先，改革力度不断加大，企业活力进一步增强。个体私营经济迅猛发展，在国内生产总值中的比重由1997年的19％增加到34％。党政机构改革任务全面完成，事业单位改革在全省率先启动。农村改革不断深化。粮棉流通体制改革全面推进，税费改革顺利实施，农民负担得到有效减控；乡镇区划调整工作稳步推进，撤并了一批规模偏小的乡镇和行政村；土地使用权流转步伐加快。对外开放迈出新步伐。2002年全市进出口总额达到39亿美元，是1997年的1.8倍，其中出口总额25.3亿美元，比1997年翻了一番多。五年累计实际利用外资14.8亿美元。外经工作一直在全省保持领先，2002年对外承包劳务营业额达到3.4亿美元，是1997年的3.2倍。国际交往和海外交流进一步扩大，新结国际友城3个。

　　--城乡面貌发生巨大变化。市区城市建设规模空前，老城区旧貌换新颜，新区建设全面启动，城市综合功能日益增强，城市品位有了较大提升。濠河风景区生态环境建设荣获"江苏省人居环境奖"。狼山、濠河风景名胜区进入全国4A级旅游区行列。各县（市）城和中心镇建设步伐加快，面貌日新月异。交通基础设施建设取得重大突破。干线公路网化工程基本建成，村村通公路目标提前实现。宁通高速公路、新长铁路南通段建成通车，结束了我市不通高速公路和铁路的历史；通启、盐通高速公路、宁启铁路南通段开工建设，全市人民盼望已久的苏通长江公路大桥奠基动工。港口、水利、电力、通讯等设施大为改善，服务功能显着增强。全市城市化率达到39％，比1997年提高8个百分点。

　　--社会事业全面发展。科教兴市步伐加快。五年来，省级以上重点科技产业化项目实施300多项。到2002年，省、市级工程技术研究中心（重点实验室）达到25家，省级以上高新技术企业达111家、高新技术产品达460多个。人才资源开发工程规划顺利实施。农业品种、技术、知识更新工程成效明显。教育现代化工程稳步推进，34个乡镇通过省教育现代化先进乡镇验收。基础教育继续在全省保持领先，职业教育、成人教育和高等教育加快发展，南通大学城建设和南通大学组建工作取得实质性进展。文化、广播电视、体育工作成绩显着。一批文艺、新闻、影视作品获国家级大奖。《南通市志》出版发行。广播电视人口综合覆盖率达100％。南通籍运动员在第27届奥运会等重大赛事上取得优异成绩，去年我市还成功申办省第十六届运动会。医疗卫生事业不断发展，初级卫生保健工作不断加强，农村改水普及率提高20个百分点。人口与计划生育工作进一步加强，人口自然增长率呈负增长。民族、宗教、侨务、台湾事务和统计、人民防空、防震减灾、无线电管理、气象、档案、保密、文博、地方志、慈善等工作进一步加强。

　　--人民生活水平明显提高。2002年，城镇居民人均可支配收入8660元，农民人均纯收入4134元，分别比1997年增长41.6％和22％；年末城乡居民储蓄余额754.7亿元，比1997年末翻了近一番。居民消费结构发生很大变化，2002年恩格尔系数为39.9％，比1997年下降11.2个百分点。住房条件大为改善，2002年城镇人均住房建筑面积24.7平方米、农村人均住房建筑面积45.3平方米，分别比1997年增加6.1平方米和10.6平方米。移动电话、电脑等现代消费品进入寻常百姓家庭。五年来共有15.3万人实现就业和再就业，城镇登记失业率始终控制在3.5％以内。下岗职工基本生活费和离退休人员基本养老金按时足额发放。城乡最低生活保障制度进一步完善，基本实现"应保尽保"。社区服务体系逐步建立。扶贫工作不断加强，平均每年扶持贫困户1.5万户，脱贫率在60%以上。妇女、儿童、老龄、残疾人和关心下一代等工作取得新成绩。安全生产呈现稳中趋好的态势。

　　--精神文明和民主法制建设进一步加强。群众性精神文明创建活动蓬勃开展，公民思想道德建设得到加强，涌现出江海志愿者等一批具有时代特点的先进典型，"送温暖、献爱心"蔚然成风，精神文明建设"南通现象"引起全国关注，被评为"省级文明城市"和"全国文明城市创建工作先进市"。军民共建、优抚安置、征兵等工作不断加强，再获"全国双拥模范城"称号。依法治市全面推进。普法工作广泛开展，公民法治意识进一步增强。市政府和各部门自觉接受法律监督和民主监督，认真执行人大决议，认真办理人大代表议案、建议和政协委员提案。民主党派、工商联和各界人士参政议政的作用得到充分发挥，公民知情权进一步扩大。与工会、共青团、妇联、科协等群众团体的联系日益密切。信访渠道进一步畅通，民调工作得到加强，廉政建设和反腐败斗争向纵深推进，惩处了一批腐败分子。"严打"斗争成绩突出，与"法轮功"等邪教组织的斗争取得重大成果，全市社会政治稳定，被评为"全国社会治安综合治理先进市"。整顿和规范市场经济秩序取得阶段性成果。

　　五年来，我们坚持以发展为主题，以结构调整为主线，以改革开放和科技进步为动力，以提高人民生活水平为根本目的，大力实施经济发展战略，狠抓各项工作落实。特别是近年来，根据不断变化的形势和任务，我们更加注重从全局性、根本性和长远性的工作着眼，从现阶段最具牵动性、突破性和基础性的事情着手，着力突破重点和难点。

　　（一）猛攻投入，增强发展后劲。把加大投入摆在突出位置，加大组织力度，完善激励政策，建立重大项目办公日和领导挂钩联系制度，组建市项目信息中心、投资促进中心，加强投入服务工作。坚持外资、民资、股资、银资和政府资金"五资并进"，市场配置与政府引导相结合，融资渠道多元化的投融资体制初步形成，2002年完成全社会固定资产投资310亿元，比上年增长20.1％。实施市重点建设项目38个，一批"双高一优"、科技计划和国债项目落户我市，同时储备了一批重大项目。银行贷款规模扩大，2002年新增贷款93.9亿元，接近当年新增居民储蓄存款。2家企业成功上市，吸纳社会资本6.3亿元。切实加强各级各类开发区、工业园区、科技产业化基地建设，载体功能迅速增强。经不懈努力，南通出口加工区获国家批准设立并封关运作。国家火炬计划南通化工新材料产业基地、中国长江国际化学纤维工业园等特色工业园区动工兴建。

　　（二）强攻改革，增创体制优势。大力推进市场取向改革，着力解决制约经济发展的体制性障碍。在以产权制度改革为核心的企业改革大力推进并取得显着成效的同时，不失时机地拓宽改革的领域。行政管理型和生产经营型事业单位转制改企取得突破性成果。以社会化、多元化和产业化为方向，推进社会事业改革，组建了中医医疗集团，乡镇卫生院改制面达到90％。在全国率先实行了住房分配、拆迁安置和经济适用房补贴的货币化。全面推行土地有偿使用制度，土地市场化供应机制进一步完善。南通港实现属地管理和政企分开。营造宽松环境，放宽发展领域，落实激励政策，大力度推进非公有制经济发展。至2002年底，全市私营企业达2.2万家，注册总资本达159.5亿元，分别是1997年的3.5倍和6倍。积极探索国有资本管理体制改革，建立市国有资产管理委员会，对7家资产经营公司实行授权经营，国有资产营运效率大大提高。加强社会保障体系建设，建立健全了养老、失业、医疗、工伤、生育等五大保险体系，覆盖面从原来的国有、城镇集体企业向外商投资企业、私营企业和个体工商户、自由职业者拓展。

　　（三）扩大开放，加快经济国际化进程。适应我国加入世贸组织的新形势，采取积极应对措施，主动融入经济全球化大潮。紧紧抓住国际资本和产业加速向长江三角洲转移的重大机遇，精心构筑招商引资新平台，组织港口经济洽谈会，实现了利用外资的突破。2002年合同利用外资、实际利用外资分别比上年增长92.6%和86.1%。利用世界经济结构调整的有利时机，大力培育精细化工、电子信息等新兴产业，改造和提升纺织服装等传统产业，加快发展城郊型、外向型农业，增强了参与国际市场竞争的能力。充分发挥江海岸线资源优势，明确了加快发展港口经济的新目标，推出了深入实施"以港兴城"战略的新举措，一批以港口为核心的重量级项目加速推进，临港产业加速催生。把上海作为走向世界的重要平台，加快接轨上海步伐。充分发挥毗邻上海的区位优势，通过多种形式推动了与上海在观念、体制、产业、环境等方面的接轨。

　　（四）提升形象，改善创业和人居环境。把优化创业和人居环境作为推动经济发展的关键措施来抓，加大力度，重点突破。全面优化创业软环境。以"三个有利于"为标准，清理地方性行政措施，制定完善吸引外资、民资和技术、人才等一系列激励政策。进行了两轮行政审批制度改革，精减行政审批事项，实行集中审批，市级审批总事项减幅近50％。各级政府和部门强化亲商意识，积极为各类业主和科技人员解难题、办实事，激发了他们的投资创业热情。大力改善城市环境。在实现届初提出城市建设"年年见成效、三年换新貌"目标的基础上，加快实施城市化战略。高起点规划城市，建立城市规划委员会和咨询委员会，邀请国内着名院校和单位参与规划设计，城市规划水平有了较大提高。高标准建设城市，拉开大城市发展框架，完善基础设施，打造"水清、岸绿、景美、游畅"的城市新景观，濠南路、濠东绿苑和环西文化广场等一批精品工程成为南通人引以自豪的城市名片。高水平管理城市，实施城市管理行政执法体制改革，进一步完善"两级政府、三级管理、四级网络"的管理体制，加大执法管理力度，市容环境有了较大的改观。切实加强环境和资源保护工作，"国家级生态示范区"建设初见成效，城乡生态环境进一步改善。

　　（五）与时俱进，加强政府自身建设。以"创新、规范、高效、廉洁"为目标，切实加强政府自身建设。坚持把开拓创新作为组织政府工作的主旋律和着力点，贯穿于各项工作之中，努力提高组织程度，加大工作落实力度。高起点确定工作思路，推动了外资热、民资热、上市热、改革热和城市热的形成，营造了"发展大升温、投入大突破、改革大推进、环境大整治"的浓烈氛围。综合运用经济、法律、行政手段调节经济，增强驾驭市场经济的能力。修改、完善《市政府工作规则》，坚持依法行政，全面推行行政执法责任制和政务公开，政府行为进一步规范。将目标责任制管理作为推动各项工作落实的重要抓手，狠抓目标分解、督促检查、考核奖惩三个关键环节，形成比较科学的目标管理和考评体系，有力地推动了机关各项工作的开展，促进了重点工作的落实。坚持科学民主决策，广泛开展人民建议征集活动，完善研究咨询网络，经常举办由国内外知名专家、学者主讲的报告会和论坛，提升了政府决策水平。开展以"服务经济建设、营造一流环境"为主题的行风评议活动，建立行政效能监察和投诉中心，实行行政过错责任追究，机关作风有了较大转变。认真落实党风廉政建设责任制，严格执行廉洁自律各项规定，强化经济审计监督，有效地探索了廉政建设的治本之策。

　　各位代表，过去的五年，是全市上下思想观念和精神面貌发生深刻变化的五年；是改革开放迈出新的步伐并取得重大进展的五年；是国民经济快速发展，人民群众得到实惠最多的五年；是城市面貌变化最大，南通形象在省内外显着提升的五年；是各项事业协调发展，社会全面进步的五年；是政府自身建设全面加强，行政效能明显提高的五年。回顾五年特别是近年的工作，我们深深地感到：高举邓小平理论伟大旗帜，贯彻"三个代表"重要思想，高扬发展主旋律，是各项事业不断从胜利走向胜利的根本；与时俱进，开拓创新，确定并坚定不移地实施切合南通实际的发展战略和工作思路，是实现富民强市目标的关键；顺民意，聚民力，得民心，调动一切积极因素，是做好各项工作的法宝。五年的发展令人鼓舞，五年的成就催人奋进。它是全市上下同心同德、苦干实干的结果，凝聚着广大创业者、劳动者的心血和汗水，展示了南通人民勇于创新、开拓进取的时代风采。在此，我谨代表市人民政府向辛勤工作在各行各业的广大干部群众，向给予政府工作大力支持的市人大代表、政协委员和离退休老同志，向关心、支持南通建设的部、省驻通单位和解放军、武警官兵及社会各界人士，向积极参与南通发展的港澳台同胞、海外侨胞和国际友人，表示崇高的敬意和衷心的感谢！

　　在肯定成绩的同时，我们清醒地看到，政府工作中存在不少薄弱环节，在前进的道路上还有不少矛盾和问题。主要是全市经济发展的速度还不快，与发达地区的差距仍在拉大；体制性障碍尚未根本消除，结构性矛盾仍然较为突出；城镇化步伐不够快，农民收入增长缓慢，农村经济发展不平衡状况仍较严重；就业和社会保障工作难度增加，可持续发展压力加大；政府职能转变和机关作风建设与形势发展要求尚有不小差距，少数干部身上还存在着消极腐败现象。这些矛盾和问题需要高度重视，并采取有效措施认真加以解决。在此，诚恳欢迎各位代表和各界人士提出批评、意见和建议。

　　二、今后五年的目标任务

　　今后五年，是全面建设小康社会，提高南通区域经济竞争力，实现跨越式发展的关键时期。今后五年，我们面临千载难逢、不可错失的历史性机遇。国际资本和产业正加速向长江三角洲转移、长江三角洲地区联动发展、上海成功申办世博会，为我们在更高起点上加快发展提供了新契机；苏通长江大桥的兴建，崇启、崇海过江通道的规划实施，高速公路、铁路、沿江港口等重大基础设施的建设，出口加工区的封关运作，为南通新世纪的加速发展打造了新平台；一批具有战略意义的重量级项目正在加紧推进，必将为南通跨越式发展蓄积新能量。同时，我们将面对国际经济环境变数更大、地区之间发展竞争更为激烈的新挑战。总的看，挑战与机遇并存，机遇大于挑战。我们要审时度势，抢抓机遇，在应对挑战中把握主动，在扬长避短中增创优势，在超越自我中寻求突破，以新的作为奋力实现跨越式发展。

　　今后五年政府工作的总体要求是：在省委、省政府和市委的正确领导下，以邓小平理论和"三个代表"重要思想为指导，认真贯彻党的十六大精神，紧紧围绕提前实现全面建设小康社会、加快向现代化迈进的目标，突出加快发展主题，贯穿结构调整主线，深入实施"四大战略"，依托江海，崛起苏中，融入苏南，接轨上海，走向世界，把南通初步建设成为经济科教发达、民主法制健全、城市功能完善、生态环境良好、人民安居乐业的现代港口、工业、贸易、旅游城市。

　　今后五年的目标建议：

　　--基本形成与人口大市相适应的经济规模和综合实力。国民经济持续、快速增长，综合实力和服务功能显着增强。发展速度和质量均高于全省平均水平，GDP、财政收入、全社会固定资产投资力争年均分别增长11％、12％、15％以上。建成有力支撑经济发展的新型产业体系，全面实现产业升级，增强持续发展能力。到2007年，人均GDP达到19000元，成为江苏和沿海城市经济增长较快的地区之一。

　　--基本形成与工业化进程相适应的城市化水平。建成强力支撑经济发展的现代化基础设施体系，完善与上海都市圈副中心城市相适应的城市功能和城镇体系，基本形成百万人口大城市的新框架和城乡协调发展的新格局。巩固"全国科技进步先进城市"，成为"国家卫生城市"、"国家环境保护模范城市"、"中国优秀旅游城市"，成为长江三角洲发展活力足、投资引力强、人居环境佳的城市。

　　--基本形成具有较高文明程度的社会发展体系。贯彻发展先进文化的要求，文化、教育、体育、卫生等各项社会事业有较大发展。自然科学和社会科学研究取得新进展，科技进步对经济增长的贡献率进一步提高。人才培养和引进取得重要成效，形成广纳群贤、人尽其才、才尽其用、充满活力的用人机制。全面加强社会主义精神文明建设，努力建设"学习型"社会，市民的思想道德素质、科技文化素质、法律素质有较大提高，基本达到与现代化进程相适应的社会文明程度，成为"全国文明城市"、全省长江以北第一个文明城市群。

　　--基本形成让人民群众得到更多实惠的保障体系。城乡就业渠道进一步拓宽，社会就业比较充分，农村劳动力转移取得新的突破，城镇登记失业率控制在4.5％以内。人民群众收入进一步提高，城镇居民人均可支配收入和农民人均纯收入年均递增8％、5％以上，高于全省平均水平。社会保障体系和社会救济制度健全，弱势群体基本生活得到保障。人民群众健康素质明显提高，社会发展转向提高人民生活质量和追求社会全面进步的新阶段。

　　展望未来，任重道远。今后五年政府工作必须把握以下几点：

　　始终保持与时俱进的精神状态，推动各项工作创新。坚决冲破思想观念上的"长江天堑"，从那些不合时宜的观念、做法和规定中解放出来，大力破除长期计划经济体制下形成的思维定势，大力破除一切从本本出发、墨守成规的僵化思想，敢于突破前人，勇于超越别人，强化小进则退的忧患意识和敢为人先的进取意识，倡导奋发有为的拼搏精神，确立矢志超越的更高追求，跳出南通看南通、比照苏南看南通、立足全国看南通，更加清醒地看到南通经济与发达地区的差距，南通工作与市场经济要求的差距，以解放思想的更高要求推动各项工作打开新的局面，以思想解放的创新举措，全面推动制度创新、科技创新、文化创新以及其他各方面的创新，为南通的发展不断注入新的活力。

　　始终把发展作为第一要务，加快富民强市进程。必须更加突出经济建设这一中心，坚持加快发展不动摇，咬住发展目标不松劲，以富民强市为第一目标，把执政兴市、执政为民作为第一追求，实现我市在苏中快速崛起，尽快融入苏南经济板块。必须找准加快发展的着力点，坚定不移地实施"科教兴市、以港兴城、经济国际化、城市化"战略和接轨上海策略，整合放大独特区位、传统产业、开放先导和人力资源四大比较优势，全面提高地区经济综合竞争力。必须全力推进重点突破，加大投入力度，通过大项目带动，高强度投入，增强经济社会长远发展的后劲和优势；加快改革步伐，深入推进市场取向的各项改革，全面创新体制机制，进一步优化所有制结构，加快市场化、民营化进程；加速结构升级，实现农业、海洋、建筑大市向强市的跨越，构建以高新技术产业为先导、基础产业和制造业为支撑、服务业全面发展的新型产业体系。

　　始终坚持"三个文明"协调发展，推进社会全面进步。围绕全面建设小康社会目标，追求经济、政治、文化协调发展。统筹城乡经济社会发展，发挥城市对农村的带动作用，加快发展市区经济，做大做强县域经济，努力解决好农业、农村和农民问题，使城市和农村相互促进、协调发展。贯彻三项基本国策，追求生产、生活、生态的和谐协调。大力加强社会主义精神文明建设，全面发展各项社会事业，培育和弘扬适应时代和发展要求的南通精神，塑造新世纪南通人的新形象。大力发展社会主义民主政治，进一步加强法制建设，大力推进依法治市，规范市场经济秩序，建设"金融安全区"，打造诚信南通。

　　始终注意最广泛、最充分地调动一切积极因素，形成凝心聚力促发展的生动局面。在市委的领导下，在市人大、市政协的监督和支持下，紧紧依靠全市人民，团结一切可以团结的力量，集中各方面智慧，全市一盘棋，上下一条心，巩固政通人和、心齐气顺的良好局面。尊重劳动、尊重知识、尊重人才、尊重创造，旗帜鲜明地为创业者壮胆、为进取者助威、为改革者护航，放手让一切发展活力迸发，让各种生产要素涌流，让广大人民群众得到更多实惠，在加快发展中启动内力，借助外力，释放潜力，形成合力，为提前实现全面建设小康社会的目标打下坚实的基础。

　　始终着力抓好政府自身建设，不断提高行政能力和服务水平。加强学习，深入调研，勇于探索，积极鼓励并投身创造性实践活动，不断提高科学决策的能力、统揽全局的能力和驾驭市场经济的能力。加快行政管理体制创新，坚持依法行政，建设高效服务型政府，更多地运用市场机制调节经济行为，运用法律手段解决社会矛盾，努力形成行为规范、运转协调、公正透明、廉洁高效的行政管理体制。牢固树立公仆和责任意识，执政为民，谦虚谨慎，艰苦奋斗，廉洁自律，不断增强政府机关和公务员的全局观念和服务经济、服务社会的责任感，深入开展"争创人民满意单位、人民满意公务员"活动，千方百计把人民群众的利益维护好、实现好。改进和完善政府工作目标责任管理，强化重点工作推进责任制，坚持领导带头狠抓落实，加强督查推动落实，严明奖惩保证落实，以良好的作风取信于民，以过硬的政绩造福于民。

　　三、2003年的政府工作

　　今年是全面贯彻落实党的十六大精神的第一年、完成"十五"计划的关键年，也是新一届政府的开局年、完成五年奋斗目标的奠基年。在开局之年起好头、迈好步，对于南通以崭新的面貌跨入新征程意义重大。立足于跨越式发展，综合考虑各方面因素，建议今年全市国民经济主要预期调控目标为：国内生产总值增长11％；财政收入增长12％以上；全社会固定资产投资增长20％；城镇居民人均可支配收入增长8％，农民人均纯收入增长5％；城镇登记失业率控制在4.5％以内；人口自然增长率控制在0.5‰以内。实现上述目标，要按照市委九届三次全体会议和市经济工作会议提出的要求，继续围绕"猛攻投入、强攻改革、扩大开放、优化环境"，重点做好十个方面的工作。

　　（一）主攻大项目，抢占产业升级新高地

　　大项目是推动产业升级，增强区域竞争力的最有效载体。要倍加珍惜、紧紧抓住世界经济结构调整和国外产业、资本大举进入长江三角洲的有利契机，牢牢盯住、全力拼抢大项目，以大项目的投入带动一大批中小项目的落户，以重点项目的突破推动产业结构的优化升级。一要突破一批拉动结构调整的大项目。着重抓好52个重点建设项目，31个重大储备项目。立足加强农业基础地位，主攻能够牵动农业产业结构战略性调整的产业化大项目，加快农业现代化进程，切实解决好"三农"问题。重点建设现代农业种养示范项目、标准化无公害项目和农副产品深加工项目，积极推广"公司加基地加农户"的产业化模式，加快土地使用权流转，加大对海安茧丝绸集团公司等国家、省级重点龙头企业扶持力度，切实抓好农产品质量体系建设，加快农业生产规模化、标准化、市场化进程。加强农业基础设施和生态建设，完成一批重点开工和续建项目，抓好中低产田改造和滩涂开发，提高农机装备水平，进一步改善农业生产条件。加大对海洋产业的投入，全面加快"海上南通"建设。立足构建现代制造业高地，主攻信息化、重化工等龙头型、基地型工业技改大项目，走新型工业化道路。年内争取有91个项目进入国家"双高一优"和省级以上高新技术项目行列。重点推进100个投资超2000万元的工业项目，加快集成电路封装等重点续建项目的建设速度，开工建设醋纤四期、宝日制钢等25个投资超亿元的项目和大生集团、中天科技等6个国债项目。坚持以信息化带动工业化，以工业化促进信息化，启动300家骨干企业的信息化建设工程。立足提升城市竞争力，主攻能够凸现区域竞争优势的大项目。加快天生港电厂扩建项目推进进程；争取如东LNG、吕四港电厂、海门电厂、市开发区JOP等投资超百亿元特大型项目正式签约、立项；营造饮江排海功能，扩大环境容量，放大江海优势。立足建设建筑强市，加大建筑企业投入，提高科技装备水平，增强承揽大型、特大型工程及其配套工程的能力，放大建筑产业整体优势。二要创新大项目的投入机制。深化投融资体制改革，对企业用自有资金及商业银行贷款建设的竞争性项目，逐步实行登记备案制。进一步拓宽社会投资领域，推进经营性基础设施和经营性社会事业向社会投资主体开放。引导外资、民资、银资、股资更多地投向新兴产业、高新技术产业和现代商贸服务业，鼓励外商特别是跨国公司在我市设立生产制造基地和研发中心。畅通多元投资渠道，千方百计扩大投资规模。打造招商平台，精心组织好'2003南通港口经济洽谈会和各种行之有效的招商活动，争取在引进外资上取得更大突破，实际利用外资增长50％左右；全方位启动、吸纳市内外民间资金；积极向上争取更多的国债、专贷、科技项目等政策性资金；强化银企合作，培植有效信贷载体，打击恶意失信行为，扩大信贷规模，缩小存贷差；建立健全中小企业信用担保体系，扩大规模，增强功能。三要提高主攻大项目的组织程度。全面落实项目推进责任制，实行项目进度绩效考核，明确奖惩措施，推动各地加大投入。鼓励和引导全市广大干部群众发挥聪明才智，实行全员招商引资，并对招商引资中成绩显着的个人给予重奖。坚持重大项目办公日制度，完善领导挂钩联系制度，确保重大项目的顺利实施。充分发挥市项目信息中心、投资促进中心的作用，加强项目跟踪，健全重大项目信息的收集、处理和反馈制度，广泛、及时、有效地捕捉项目；建立项目推进投诉制度，将服务项目建设作为行政效能监察的重点，对影响项目招商、立项和建设的部门和工作人员进行严肃查处。

　　（二）构建大园区，提升开放开发新水平

　　经济发展潜力在园区、希望在园区。必须把园区建设放到更加突出的位置，着力构建以南通经济技术开发区为龙头、省级开发区为主体、中心镇工业园区为支撑的园区群。一要科学规划，合理布局。立足于增强区域整体竞争力，加快制定新一轮园区建设规划，明确各级各类园区的区划、定位和发展方向，做到布局合理、错位发展、共同繁荣。重视对现有园区的整合，引导各类企业和项目向园区集中。园区建设既要积极推进，又要注重实效，尽力提高基础设施共享度，防止土地、基础设施等资源的闲置与浪费。二要加快开发，增强功能。进一步增强各类园区的要素集聚、科技创新、产业吸纳功能，使之成为对外开放的大窗口、招商引资的主阵地、项目投入的集中区。同时，注意发挥各类园区对面上的示范、辐射和对城镇建设的带动作用。拓宽筹资思路，加快各级各类园区的基础设施和配套设施建设步伐，全面提高硬件水平。制定出台招商项目跨区投入的税收分成、指标统计政策，促进各类项目到本市适宜的园区发展。以更到位的服务和更灵活的机制，营造区域经济发展的特区。三要分类推进，打造特色。加快出口加工区、长江国际化学纤维工业园、长江国际化学新材料工业园、国际电子模具园、留学人员创业园、综艺数码城和俄罗斯高科技成果转化园等特色园区的建设速度，尽快形成化纤化工、电子、模具等产业新高地。加强对全市各类农业示范园区资源的整合，加快现代农业产业园区建设步伐。每个县（市）区建成1个万亩以上的市级现代农业产业园区。注重乡镇工业园区规模化、特色化建设，力争营业收入超5亿元乡镇工业园区达到15个，创建省级示范园区4个。

　　（三）培育大主体，增添经济发展新动力

　　增强区域经济的竞争力，必须遵循市场经济的客观规律，加快培育各类充满活力的市场经济主体。一要不失时机地大力推进国有经济的战略性调整，进一步激发企业活力。以更高的目标，更大的力度，更实的措施，组织市县属企业改革的大会战，确保市属企业"两退"任务基本完成，县属企业改革全面做好扫尾工作。今年公有资本退出要从中小企业向其他企业拓展，除极少数暂需保留国有资本外，凡正常经营的企业公有资本年内必须全部退出，劣势企业除特殊情况外年内必须全部退出市场。深化国有资产管理体制改革，完善市级国有资产经营公司的运行机制，拓展国有资产授权经营范围，推进国有经济布局的调整，确保国有资产保值增值。坚持市场化改革方向，加快推进社会事业改革，公益型事业单位分类改革试点抓紧推进，生产经营型事业单位上半年完成改制任务。二要不失时机地大力推进非公有制经济发展，充分释放个体私营经济的巨大增长潜力。适应民营化的大趋势，抓住机遇，乘势而上，保持个体私营经济高速增长的良好势头，把个体私营经济培育成具有强大活力的主流经济，力争个体私营经济占GDP的比重比上年增加5个百分点。按照"六放"要求，落实鼓励个体私营经济发展的政策措施，营造更为宽松的环境，最大限度地拓展个体私营经济发展的空间。积极推动个体私营企业做大规模、提升层次，重点扶持一批规模型、科技型和外向型的私营企业。三要加速培育骨干企业，切实增强核心竞争力。认真贯彻市政府《关于加快培育和发展具有国际竞争力的重点大企业（集团）的意见》，坚持政府推动、市场化运作、企业自主决策的原则，积极推动优质资产、存量资产向优势企业集聚，着力培育主业突出、技术先进、竞争力强的大企业、大集团，力争年内有3－4家企业进入上市辅导期，1－2家企业成功上市，4－5家重点企业营业收入超过30亿元，其中文峰集团突破50亿元。

　　（四）构筑大通道，凸显区域竞争新优势

　　加快构筑迅捷流畅的大通道，消除长江阻隔，对加速融入上海都市圈，增强区域竞争优势，具有决定性意义。一要加快我市对外通道建设步伐。在加快建设苏通大桥的同时，积极推进沪通铁路和沪崇启、崇海过江通道规划建设，沟通南通连接上海和苏南的大通道。抓紧实施通启高速公路、盐通高速公路和干线公路网化工程以及港口连接线、农村公路工程。加快推进宁启铁路海安段建设，启动实施狼山港铁路支线建设。抓紧改善汽渡渡运条件，增加渡轮班次，缩短待渡时间。二要加快培育通道经济。借助大交通，发展大产业。充分依托骨干道路交通网络沟通四面八方的便利条件，超前制定沿交通干线和交通道口周边区域的产业发展规划，合理规划用以发展各类载体的用地空间，培育壮大沿路轴线经济和道口经济，形成集聚要素的强大磁力，争创区域经济发展新亮点。抓紧做好苏通大桥北桥头区的规划，明确功能布局与发展时序，使之尽快成为接受产业转移的前沿阵地和汇聚国内外人流、物流、信息流、资金流的重要节点。三要加快接轨上海的步伐。出台并实施《南通市接轨上海实施纲要》，建立接轨工作机构，开展对接交流活动，增强承接上海辐射能力，提高服务上海水平，从思想观念、产业发展、生产要素、体制机制、制度政策等方面全面加快与上海对接。抓住上海成功申办世博会、实施黄浦江两岸综合开发的机遇，充分发挥我市区位优势，参与场馆建设，吸引沪企北移，加速共建沪通工业走廊进程，在主动服务中捕捉商机，在错位竞争中赢得市场份额，加紧把南通建成上海的农副产品生产基地、加工工业生产基地、特色旅游休闲基地和建筑业服务基地。在实施接轨上海的同时，大力加强与长江三角洲城市及区域特别是苏南地区的合作，联动发展产业，联动开发园区，联动建设基础设施，促进区域经济共同发展。

　　（五）建设大港口，营造江海共兴新格局

　　依托得天独厚的港口条件和丰富的岸线资源，强化沟通东西、连接南北、大进大出的"江海桥头堡"功能，加快沿江沿海港口开发进程，尽快形成江港与海港共兴的生动局面。一要整合资源，构筑沿江港口集群优势。高起点编制沿江发展整体规划，合理确定港口岸线、工业岸线、生态岸线和城市生活岸线。对现已开发利用的岸线和泊位资源积极实施有序整合。以长江深水泊位的开发建设为重点，加快老港区改造升级，加快狼山港三期工程的实施，加快如皋港等新港区的开发建设，做好通海港区开发的前期工作，尽快形成南通港重量级泊位的新优势。充分借助上海国际航运中心的优势，加强与国内外船务公司的合作，增辟远洋航线，提高航班密度，进一步提升港口集疏运功能。二要项目带动，加快启动海港建设。在做大沿江港口的同时，不失时机地加快海港开发进程，市县联动，合力突破项目前期工作，以大项目带动快开发。三要依托江海，加快发展临港产业。依托和发挥港口优势，加快推进沿江、沿海产业带建设，引进发展大运量、大用水的临港吞吐型和饮江排海型、产业集聚型、出口加工型工业项目，加快南通开发区港口工业一区、二区、三区建设，规划建设如东、启东、如皋、通州、海门港口工业区，进一步优化生产力布局。

　　（六）发展大流通，开拓资源配置新领域

　　顺应经济全球化的新形势和要素流通加快、消费需求升级的新趋势，充分利用国际国内两个市场，优化资源配置，拓展发展空间。一要内外并举搞活大贸易。以提高水平、优化结构为重点，突出发展新型、新兴商贸流通业，大力发展连锁经营、物流配送等新型业态，着力建设具有中心集聚效应的大型商贸区，年内建成7个商贸中心区、8条专业特色街、9个社区商贸服务中心。以搞活农村市场、农产品流通为突破，大力发展各类农村合作经济组织、专业协会、农民经纪人和营销大户，进一步提高农民进入市场的组织化程度。继续做好入世应对工作，加快质量认证步伐，有效规避和破解"绿色壁垒"、"技术壁垒"，大力实施出口"以质取胜、市场多元化"战略，调整外贸商品结构和市场结构，优化外贸经营环境和口岸通关环境，不断完善税贸、关贸、检贸、银贸协作机制，扶持外贸出口大户，培植外贸出口新户，开拓新兴市场领域，自营出口额增长11％。大力实施"走出去"战略，发挥我市劳动力素质高的优势，充分挖掘潜力，培育壮大外经主体，拓展外经市场，加强规范管理，保持在全省的领先位置，对外承包劳务营业额增长11％。二要提升功能完善大市场。继续搞好商品市场建设，促进和加快大市场的形成，提升市场形态。抓好市区农贸市场的升级改造。深化粮棉购销市场改革，搞好粮油批发市场和棉花交易市场建设。培育消费热点，拉动消费需求，社会消费品零售额增长11％。利用上海等地要素平台，进一步培育发展人才、科技、信息、产权等要素市场，促进生产要素合理流动，在更大程度、更高层次上发挥市场在资源配置中的基础性作用。充分利用金融市场和资本市场，加快投融资市场化步伐。切实加强市场管理，继续整顿和规范市场经济秩序，推行市场准入制度，巩固和扩大市场"肉菜粮放心工程"成果。三要多方协力构建大物流。以港口为枢纽，产业发展为依托，抓紧制定实施我市现代物流业发展规划，促使南通早日成为长江三角洲北翼的区域性物流中心。要合理确定沿江港区功能与布局，着力推进大宗物品、集装箱、化学品3大物流园区的规划建设，先期发展液体化工、钢材、油品3大配送中心。积极发展第三方物流，推动传统交通运输业向专业化、社会化的物流企业的转型，重点培育1-2个上规模、有竞争力的现代物流企业。加快建设物流信息平台，加快口岸直通式海关监管点建设，实施大通关、快通关，实现港务、口岸、电信、海关、国检、税务、银行等单位联网，沟通货物流、信息流、资金流，EDI物流信息系统确保在年内投入运行。

　　（七）开发大旅游，培植经济增长新亮点

　　发挥我市人文资源丰富、江海风情独特等比较优势，通过体制、机制的突破，做大做强旅游产业，使之成为强劲的经济增长点。一要加快整合旅游资源。科学制定和实施南通旅游发展总体规划，明确我市旅游的发展方向、产业定位和功能布局，实现旅游资源的合理开发和优化配置。进一步理顺管理体制，强化发展大旅游的工作机制，有效解决资源分割、多头管理的突出问题。积极开发主题旅游，把旅游资源与旅游产品、产业串联起来，把南通的各种优质旅游资源整合起来，放大旅游优势，提高旅游产业效益；深度开发特色旅游，充分挖掘佛教名山、江海情韵、田园风光、青墩遗址、养生文化内涵，搞好重点景区和旅游项目建设，发展以"五山、濠河"为核心的宗教、休闲、观光、生态等江海风光特色旅游，重点抓好濠河周边文博馆群、五山整体开发、如皋长寿文化园、通州开沙岛、海门风情园、东灶蛎蚜奇观、如东"海上迪斯科"、启东圆陀角等项目建设。在巩固两个4A级景区的基础上，抓紧培育3-4个3A级景区景点，形成江海旅游支撑骨架。二要加快培育旅游开发、经营市场主体。加强政策引导，研究制定扶持旅游业发展的政策措施，加大对旅游业的投资力度，催生更多的旅游开发、经营主体；加快机制创新，增强发展活力，变部门直接经营为企业开发经营，通过市场化运作、专业化招商、产业化发展，吸引更多的社会资本、民间资本参与旅游资源开发，真正搞活经营机制。三要铸造江海旅游品牌。围绕打造"中国近代第一城"，精心组织包装，加大宣传推介和引资开发力度，提高旅游市场竞争力。组织富有地方特色的旅游节，充分利用新闻媒体、网络传媒、节庆活动，叫响"追江赶海到南通"主题口号。深入开展创建"中国优秀旅游城市"活动。加强旅游市场管理，搞好导游队伍建设，以优良的市场秩序和优质的导游服务，树立南通旅游的崭新形象。

　　（八）打造大城市，拓展城乡建设新空间

　　按照建设长江三角洲人居环境最佳的生态城市、上海北翼的现代化港口、工业、贸易、旅游城市的定位，加快城市化进程，全面提高城市发展质量和城镇化水平，实现城乡共同发展。一要做大中心城市，增强集聚功能。以建设百万人口大城市为目标，调整完善城市总体规划，加快编制港闸区、苏通大桥北桥头区等分区规划以及各类详细规划，高起点搞好通吕运河两岸建设规划。按照"分类指导、重点突破、整体推进、协调发展"的总体思路，充分调动市、区两级的积极性，继续推进老城区改造，全面加快新区、狼山风景区、港闸区、南通开发区、崇川观音山区域的建设步伐，扎实推进重点道路建设工程，进一步拉开城市发展框架。打造城市建设精品，提升城市品位。高起点、高标准建设体育广场、新区行政中心等一批标志工程，启动体育、会展中心建设，再造城市建设新"亮点"。以建设园林城市为目标，绿化、净化、美化城市环境，提升城市形象。加快实施绿化工程，提高城市沿道、沿河、沿街绿化覆盖率；对204国道市区段、外环北路、疏港路等进入市区的主要通道进行"美容"，改变临道环境面貌；强化以交通、卫生、市容环境、市场秩序、小区管理等为重点的城市综合管理，拓展城市综合服务功能。二要创新经营城市理念，增强"造血"功能。不断加大土地供应市场化配置程度，提高城市空间资源经营水平，增强城市建设积累能力。构筑城建项目融资平台，通过市场化运作，推动外资、民资和社会资金投入城市建设，不断扩大城建资金总量规模。三要促进城乡共同繁荣，实现可持续发展。加快县（市）城和省级重点中心镇基础设施建设，提高规划水平，提高建设档次，促进产业集聚。深化城镇户籍等制度改革，加速农村富余劳动力向非农产业和城镇转移。认真贯彻计划生育、保护环境和保护资源的基本国策。稳定低生育水平，提高人口素质，全面争创计划生育优质服务先进县（市）区和省计划生育工作示范、先进县（市）。进一步改革土地使用制度，合理开发和节约使用各种自然资源。继续推进区域供水工程，做好通启、通吕运河等流域污染治理工作，完成濠河、红庙子河污水截流工程，加快市区污水处理基础设施建设，全面改善生态环境，争创"国家环保模范城市"。

　　（九）繁荣大文化，实现社会事业新跨越

　　牢牢把握先进文化的前进方向，努力建设富有时代特征和南通特色的文化大市，为加快经济社会发展提供方向先导和智力支持。一要以文化产业的大发展，带动文化事业的大繁荣。积极探索新的文化产业发展机制，鼓励和引导社会资本与国际资本进入文化产业，通过市场化改革、产业化整合，推动文化产业升级、文化事业单位转型。完善和落实文化产业政策，构建以现代传媒业、新闻出版业、文化旅游业、体育产业等文化重点产业为主导、相关产业联动发展、结构优化的文化产业体系。以"五个一工程"为龙头，精心组织精神文化产品生产，不断推出精品力作。积极发展文博事业，规划建设博物苑新馆等一批标志性文化设施，保护和开发好地方特色文化，彰显南通地域文化魅力，提升城市文化品位。加强和改进基层文化工作，着力抓好农村基层文化网络和设施建设。广泛开展全民健身运动，启动省十六运办赛、参赛各项工作。进一步深化卫生改革，完善医院集团化经营，加强药品监督管理，搞好药品流通体制改革，建立健全卫生服务体系和医疗保障体系。二要以科技与经济的紧密结合，推动"科教兴市"战略的深入实施。加大科技投入，加快科技创新，加强产权保护，再创"全国科技进步先进城市"。加快国家火炬计划南通化工新材料产业基地建设，滚动扶持50个重点科技项目和50家重点科技企业，推动传统产业高新技术化、国民经济信息化。大力培养和引进高层次人才，构建人才高地。巩固和提高基础教育，保持在全省、全国的领先地位。年内完成100所中小学布局调整，努力扩大优质教育资源，创建省教育现代化先进乡镇5个。积极推进南通大学城和南通综合性大学建设，大力发展职业教育、成人教育和多种形式的继续教育，构建终身教育体系。三要以强化思想道德建设为核心，大力弘扬社会主义精神文明。瞄准建成全国首批文明城市目标，巩固和深化创建工作成果，加强社会公德、职业道德、家庭美德教育，组织干部群众积极参加公民道德实践和文明社区、文明村镇、文明单位等群众性创建活动，在全社会形成爱岗敬业、乐于奉献的良好风尚，形成和睦友爱、互相帮助的人际关系，发扬光大精神文明建设"南通现象"，形成文明礼貌、遵纪守法的社会风气，进一步激发热爱南通、建设家乡的热情。继续大力加强双拥和优抚工作，支持驻通部队建设，确保蝉联"全国双拥模范城"。积极做好人民防空、防震减灾、气象和地方志等工作。

　　（十）优化大环境，增强加快发展新合力

　　环境是加快发展的第一要素，环境的优劣决定着一个地区经济与社会发展的兴衰成败。必须高度重视，着力优化社会发展环境。一要大力营造充满活力、有利发展的体制环境。立足于解放和发展生产力，解决好经济生活中存在的体制"瓶颈"，为经济发展创造良好的制度条件。进一步理顺政企、政社、政资关系，大力培育和发展行业协会、中介组织。按照决策、执行、监督相协调的要求，积极进行行政执法权相对集中探索，推行电子政务，努力提高行政效率，降低行政成本。加快社会保障体制改革，健全社会保障体系。千方百计扩大就业，认真落实各项支持就业再就业的扶持政策，逐步建立劳动者自主就业、市场调节就业、政府促进就业的新机制。进一步加大社会保险基金的征缴力度，扩大基本养老保险、失业保险、医疗保险覆盖面，全面落实三条保障线制度，巩固"两个确保"，切实保障社会弱势群体的基本利益。稳步推进农村养老、农民大额费用合作医疗保险和最低生活保障，积极搞好扶贫工作和老区开发。加强社会救助体系建设，不断提高保障水平，使全市人民共享社会文明与进步成果。深化农村改革。巩固和发展农村税费改革成果，确保农民负担不反弹，确保义务教育经费正常需要，确保基层政权正常运转。二要大力营造政策透明、服务优越的政务环境。坚持"创新、规范、高效、廉洁"，树立政府良好形象。强化亲商意识，减少审批环节，推行"并联审批"，降低投资门槛，减少商务成本，重点对审批、退税、通关、办证、年检等事关投资形象、服务效率的关键部位进行集中诊断、有效整治，对越权收费、多头收费、超标收费行为进行重点查纠，对破坏投资形象、损害发展大局的人和事，坚决彻查，实施过错责任追究。取消过时的限制性、歧视性政策，增加经济发展政策的透明度。推进公共财政改革，全面推行部门预算和国库集中支付制度，进一步落实收支两条线、经济责任审计等措施，扎实推进机关部门"三清"（清资产、清资金、清实体）工作。三要大力营造管理有序、行为规范的法治环境。进一步贯彻依法治国的基本方略，加快推动依法治市进程。继续推进社会主义民主政治建设，保障人民依法行使民主权利。自觉接受同级人民代表大会及其常委会的法律监督和工作监督，依法向人大及其常委会报告工作，切实执行人大及其常委会的决议决定；积极支持人民政协履行政治协商、民主监督和参政议政主要职能，认真听取各民主党派、工商联、人民团体、无党派民主人士意见。注重提高人大代表议案、建议和政协委员提案办理满意率。全面落实党的民族、宗教、侨务政策，支持工会、共青团、妇联、科协、社科联、残联等群众团体工作。扩大基层民主，推行村民自治，完善职工代表大会等民主管理制度。深入开展"四五"普法教育，提高公民法律素质。坚持依法决策、管理经济和社会事务，提高政府机关依法行政水平。认真贯彻统计、保密、档案等法律法规。加强老龄和关心下一代工作，切实维护好妇女、老年人、未成年人合法权益。强化社会治安综合治理，继续深入开展严打整治斗争，防范和惩治邪教组织的犯罪活动，坚决扫除社会丑恶现象。高度重视并正确处理新形势下人民内部矛盾，认真做好信访工作，切实落实责任制，积极预防和妥善处理群体性事件，努力化解不稳定因素。加强安全生产，深入推进专项整治，大力消除事故隐患，杜绝重特大事故发生，创造安定的社会环境。

　　各位代表，新的形势催人奋进，新的事业需要我们奋力开拓。我们必须倍加珍惜机遇，倍加顾全大局，倍加珍视团结，聚精会神搞建设，一心一意谋发展。让我们高举邓小平理论伟大旗帜，全面贯彻"三个代表"重要思想，沿着党的十六大指引的方向，在省委、省政府和市委的正确领导下，紧紧依靠和带领全市人民，团结一心，励精图治，与时俱进，开拓创新，全面开创我市改革开放和经济社会发展的新局面，共同创造幸福生活和美好未来！
